# How are the portuguese coping with the mandatory confinement by COVID-19? Association between mental health and coping strategies

**DOI:** 10.1192/j.eurpsy.2021.798

**Published:** 2021-08-13

**Authors:** M. Jarego, F. Pimenta, J. Pais-Ribeiro, R. Costa, I. Patrão, L. Coelho, A. Ferreira-Valente

**Affiliations:** 1 William James Center For Research, ISPA, Lisboa, Portugal; 2 School Of Psychology And Health Sciences, University of Porto, Porto, Portugal; 3 Applied Psychology Research Center Capabilities & Inclusion, ISPA, Lisboa, Portugal; 4 Center For Social Sciences, University of Coimbra, Coimbra, Portugal; 5 Department Of Rehabilitation Medicine, University of Washington, Seatle, United States of America

**Keywords:** coping, COVID-19, mental health

## Abstract

**Introduction:**

The global COVID-19 pandemic has had an unprecedented effect on human behaviour and wellbeing. However, researchers have not yet considered how coping responses to stress related to COVID-19 could influence mental health.

**Objectives:**

This study aims to evaluate the mental health status of Portuguese during the national lockdown; examine how study participants cope with stress during the national lockdown; and assess the association between coping and mental health status.

**Methods:**

We cross sectionally analysed data from a convenience sample of 430 adults living in Portugal. Mental health was measured using the five-item Mental Health Inventory. Coping strategies were assessed using the Brief COPE. We examine the univariate associations between mental health status and coping responses. We performed a multiple hierarchical regression analysis controlling for sex and age, to test the predictive importance of coping responses on mental health status.

**Results:**

Participants’ mental health was lower than the cut-off point for poor mental health (p<.001). The use of instrumental support, emotional support, self-blame, venting, denial, behavioral disengagement, and substance use were positively significantly associated with mental health, while active coping, positive reframing, acceptance, and humor were negatively significantly associated with mental health. The multiple hierarchical regression analyses showed that sex and age accounted for 6% of the variance of mental health. Coping strategies accounted for an additional and statistically significant 30% of the variance of mental health.

**Conclusions:**

The findings provide support for the impact of the coping strategies on mental health. We encourage future research on the present topic.

